# Transcriptomic analysis of a moderately growing subisolate *Botryococcus braunii* 779 (*Chlorophyta*) in response to nitrogen deprivation

**DOI:** 10.1186/s13068-015-0307-y

**Published:** 2015-08-28

**Authors:** Lei Fang, Deying Sun, Zhenyu Xu, Jing He, Shuyuan Qi, Xin Chen, Wee Chew, Jianhua Liu

**Affiliations:** Collaborative Innovation Center of Deep Sea Biology, Ocean College, Zhejiang University, Hangzhou, 310058 Zhejiang China; Genome Institute of Singapore, A-STAR, Singapore, 138672 Singapore; Ocean Research Centre of Zhoushan, Zhejiang University, 10 Tiyu Road, Room 502, Zhoushan, 316021 Zhejiang China; Zhejiang Provincial Key Laboratory for Microbial Biochemistry and Metabolic Engineering and College of Life Sciences, Zhejiang University, Hangzhou, 310058 Zhejiang China; Institute of Chemical and Engineering Sciences, A-STAR, Singapore, 627833 Singapore; Dalian Ocean University, Dalian, 116023 Liaoning China; Biopolis Shared Facilities, A-STAR, Singapore, 138671 Singapore

**Keywords:** *Botryococcus braunii*, Green microalgae, Hydrocarbon, Transcriptome, Response to nitrogen deprivation

## Abstract

**Background:**

The colonial microalga *Botryococcus braunii* has been brought to people’s attention for its conspicuous ability to accumulate a variety of lipids including hydrocarbons. *B. braunii* strains are classified into 3 races based on the types of hydrocarbons. A and B races are known to accumulate high level of lipids. However, their extreme slow growth rate has impeded its application for renewable biofuel production.

**Results:**

In this study, we report the transcriptomic response of a moderately growing subisolate from the culture of *Botryococcus* sp. CCALA-779 upon nitrogen deprivation (ND). We show that the subisolate has an average growth rate of 0.52 g l^−1^ day^−1^ under photoautotrophic growth conditions and lipid content is enhanced to 75 % of CDW upon ND. Both rDNA sequence and hydrocarbon composition analyses indicate that the subisolate belongs to A race *B. braunii*. Hence, it is designated as *B. braunii* 779. We show that *B. braunii* 779 transcriptome shares homology to majority of the A race but not B race *B. braunii* ESTs, suggesting that transcriptomes of A race differ from that of B race. We found that many homologous ESTs between A races 779 and Bot-88 are unknown sequences, implying that A race contains many unknown genes. Pathway-based transcriptomic analysis indicates that energy metabolisms are among the top expressed functions in log-phase cells, indicating that the slow growth rate is a result that energy flow is directed to lipid biosynthesis but not population growth. Upon ND, reconfiguration of metabolisms for reducing power is apparent, suggesting that *B. braunii* 779 is rapidly adapting under ND condition by transcriptomic reprogramming.

**Conclusions:**

Taken together, our result shows that the subisolate *B. braunii* 779, similar to the Gottingen strain, is useful for biofuel production. Difference between transcriptomes of A and B races implies that different races of *B. braunii* strains belong to different sub-species. Furthermore, there are many novel genes that are unique to A race, suggesting that sequences of many enzymes involved in hydrocarbon biosynthesis are not currently known. We propose that *B. braunii* transcriptomes provide a rich source for discovery of novel genes involved in hydrocarbon biosynthesis.

**Electronic supplementary material:**

The online version of this article (doi:10.1186/s13068-015-0307-y) contains supplementary material, which is available to authorized users.

## Background

Microalgae have been getting renewed interests for its CO_2_ mitigation and lipid production. Photosynthetic microalgae, like green plants, utilize photon energy to convert CO_2_ (in presence of water) into sugar and oxygen. Lipid yield from oleaginous algae is believed to be at least an order of magnitude higher than that of many energy plants [1, 2], besides that cultivation of microalgae can avoid competing arable lands and irrigation water for crops [3, 4]. Many oleaginous microalgae accumulate triacylglycerols (or TAG) that need to be transesterified with methanol to methyl esters of fatty acids and glycerol, for example, before being refined to biodiesel [5]. On the other hand, oleaginous green colonial microalga *Botryococcusbraunii* has been brought to people’s attention for its conspicuous ability to accumulate a variety of lipids including hydrocarbons that are found in petroleum deposits and can be refined directly without the need of esterification because they are not fatty acids [4, 6].

However, *B. braunii* has suffered from an extreme slow growth (i.e., population doubling time is approximately 5–7 days) that has hampered from its application for biofuel production [6]. The slow growth has also prevented it from genetic studies. Hence, many researches have focused on the analysis of hydrocarbon compositions and the discovery of DNA sequences encoding enzymes involved in biosynthesis of hydrocarbons [7–12]. Depending on the types of hydrocarbons accumulated in cells and cell wall matrix, *B. braunii* is classified into three principal races, A, B, and L. A race produces very long chain fatty acid (or VLCFA)-derived odd-carbon-numbered *n*-alkadiene and alkatriene, B race produces mainly botryococcenes (or triterpenoids), and L race produces lycopadiene (or tetraterpenoid) [13–16]. Several genes encoding enzymes such as triterpene methyltransferases TMTs [8], 1-deoxy-D-xylulose 5-phosphate synthase DXS [10], squalene synthase-like SSL botryococcenes synthase [9, 17], Botryococcus squalene synthase BSS [18], and squalene epoxidases SQE [11] have been cloned and their enzymatic activities have been tested.

Genomic sequences of a number of photosynthetic microalgae have been completed (http://genome.jgi-psf.org). These comprehensively annotated genomes have provided rich sources for annotation of novel genomes and transcriptomes. Using the comprehensively annotated genomes, de novo transcriptome analysis without reference genomic sequences has permitted the discovery of genes and reconstitution of metabolic pathways in some non-model microalgae [19–22]. De novo transcriptomic analyses in *B. braunii* have extended our knowledge on potential enzymes involved in biosynthesis of hydrocarbons in *B. braunii* [21, 23–25]. For example, 19 out of 55 sequences encoding enzymes potentially involved in biosynthesis of VLCFA have been discovered in A race *B. braunii* Bot-88 [24] and 100 curated and machine-assembled sequences potentially involved in biosynthesis of botryococcenes and squalene have been revealed in B race *B. braunii* Showa [21]. However, it is unclear why not all the enzymes that are involved in biosynthesis of VLCFA are found in the transcriptomes of A race *B. braunii*.

ND is one of the most widely used methods for enhancement of storage lipids in a number of TAG-containing green microalgae [2, 5, 22]. In A race *B. braunii*, nitrogen limitation enhances accumulation of oleic acid [26], but not hydrocarbons [27]. Global transcriptional profiling of microalgal cells in response to ND using next-generation sequencing (or NGS) technologies allows identification of gene regulatory networks involved in adaptation and survival [22, 28, 29].

In this study, we report (1) the characterization of a moderately growing subisolate derived from the *Botryococcus* sp. CCALA779 culture, (2) biochemical analysis of the lipids and hydrocarbons accumulated in the subisolate, and (3) transcriptomic profiling of the subisolate in response to ND. We show that the subisolate can reach a maximal growth rate of 1.23 g l^−1^ d^−1^ and an average growth rate of 0.52 g l^−1^ d^−1^ in 2× BB medium [30] under photoautotrophic growth conditions. Lipid contents are enhanced by nitrogen deprivation (ND). The 18S rRNA sequence-based phylogenetic analysis places the subisolate to the group of A race. Consistent with this, GC–MS analysis of hydrocarbon composition in the subisolate reveals the presence of heptacosdiene and heptacostriene. Four giga-nucleotide short-read sequences of RNA (or cDNA) generated using Illumina sequencing platform were assembled by Trinity software. Approximately 20 % (i.e., 12,292) of the non-redundant transcriptome was found to have best-hit in the 6 algal genomes that are comprehensively annotated (http://genome.jgi-psf.org). Comparison to other previously reported transcriptomes of other *B. braunii* strains implies that the similarity between transcriptomes of A and B races is very low (i.e., at a level of 15 %). On the other hand, majority of the homologous ESTs between A races are unknown sequences. Taken together, our results indicate that the moderately growing subisolate belong to the A race *B. braunii*. Transcriptomes between A and B races *B. braunii* differ dramatically, suggesting that A and B races belong to different sub-species. Additionally, many ESTs in A race transcriptomes are unknown sequences, providing a useful resource for discovery of novel genes. We propose that the *B. braunii* 779 is similar to other well-characterized strains such as Gottingen strain [27], attractive for biofuel production.

## Results

### Accumulation of lipid and hydrocarbon in a moderate growing *Botryococcus* subisolate is enhanced by ND

**Fig. 1:**
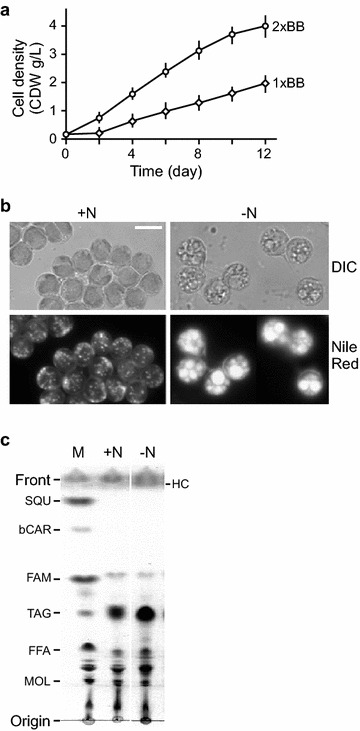
Characterization of the moderately growing subisolate from a *Botryococcus* CCALA 779 culture. **a** Growth curve of the subisolate. X- and Y-axis indicate time in days and cell dry weight in gram per liter. Nutrient strength of 1× BB and 2× BB indicates medium with standard nutrient concentration and twofold of standard concentration. **b** Fluorescence microscopic analysis of Nile red-stained cells of subisolate. Cells prior to and after ND are indicated by +N and −N, respectively. Cell images are captured under the setting of differential interference contrast (DIC) and fluorescence (Nile red). A *scale bar* of 20 μm is shown. **c** Analysis of total lipids extracted by methanol/chloroform solution (i.e., 2:1 by volume). Equal amount of total lipid extract from cells prior to (+N) and after (−N) ND is loaded onto a TLC plate and developed with hexane/diethyl ether/acetic acid solution (i.e., 35:15:0.1 by volume). Origin and front of TLC are indicated. Marker (M) of standard chemicals includes squalene (SQU), beta-carotene (bCAR), FAME C_14_-C_22_ (FAM), tri-oleic acid glyceride (TAG), oleic acid (FFA), and mono-olein (MOL). *HC* hydrocarbon.

A moderately growing subisolate from a culture of *Botryococcus* sp. CCALA 779 on solid BB medium was obtained. Photoautotrophic growth rate of the subisolate in liquid BB medium was examined under the condition of 250 μmol photon m^−2^ s^−1^. The growth curve indicated that this subisolate exhibited a maximum growth rate of 0.61 g l^−1^ d^−1^ (i.e., gram of cell dry weight or CDW per liter per day) and a maximum cell density of 1.98 g l^−1^ in terms of CDW per liter (Fig. 1a; Table 1). When using 2× BB medium (i.e., to double the nutrient concentration of the BB medium), we found that both the growth rate and cell maximum density were doubled to 1.23 g l^−1^ d^−1^ and 4 g l^−1^, respectively. Subsequently, we examined the content of lipid and hydrocarbon in log-phase cells (e.g., at the density of ~1.61 g/L in 1× BB medium) that were extracted by methanol/chloroform solution (i.e., the ratio was 2:1 by volume) and hexane, respectively, using gravimetric methodologies (see “Methods”). Our results indicated that the total lipid and hydrocarbon contents in log-phase cells were approximately 0.58 g/L (or 36 % of CDW) and 0.31 g/L (or 19 % of CDW), respectively (Table 2).

**Table 1:** Growth curves of the *Botryococcus* sp. subisolate

Time	CDW1^a^	CDW2	CDW3	CDW (Ave ± SD)^b^
(A) In BB medium				
0 (d)	0.140	0.155	0.155	0.150 ± 0.009
2	0.215	0.245	0.235	0.232 ± 0.015
4	0.600	0.640	0.630	0.623 ± 0.021
6	0.920	0.960	0.970	0.950 ± 0.026
8	1.150	1.350	1.300	1.267 ± 0.104
10	1.500	1.700	1.650	1.617 ± 0.104
12	1.900	2.200	1.900	2.000 ± 0.173

Period	Rate1^c^	Rate2	Rate3	Rate (Ave ± SD)
(A) In BB medium				
0–2	0.239	0.257	0.231	0.243 ± 0.013
2–4	0.671	0.616	0.637	0.641 ± 0.027
4–6	0.238	0.225	0.241	0.235 ± 0.009
6–8	0.118	0.186	0.158	0.154 ± 0.034
Average growth rate	0.318			
Maximum growth rate	0.641			

Time	CDW1	CDW2	CDW3	CDW (Ave ± SD)
(B) In 2× BB medium				
0 (d)	0.140	0.155	0.155	0.150 ± 0.009
2	0.730	0.755	0.750	0.745 ± 0.013
4	1.610	1.580	1.630	1.607 ± 0.025
6	2.250	2.500	2.550	2.433 ± 0.161
8	3.000	3.000	3.300	3.100 ± 0.173
10	3.500	3.850	3.850	3.733 ± 0.202
12	3.800	4.300	4.050	4.050 ± 0.250

Period	Rate1	Rate2	Rate3	Rate (Ave ± SD)
(B) In 2× BB medium				
0–2	1.2835	1.2070	1.1997	1.2301 ± 0.0464
2–4	0.4851	0.4466	0.4742	0.4686 ± 0.0198
4–6	0.1822	0.2579	0.2508	0.2303 ± 0.0418
6–8	0.1547	0.0954	0.1376	0.1292 ± 0.0305
Average growth rate	0.515			
Maximum growth rate	1.230			

^a^CDW stands for cell try weight, repeat number is indicated.

^b^Ave and SD stand for average and standard deviation based on 3 repeats.

^c^Growth rate in an 2 days-period of 0–2 days, 2–4 days, 4–6 days, and 6–8 days was calculated using the formula $\sqrt[2]{CDW_{t2}/CDW_{t1}}- 1.$

**Table 2:** Lipid and hydrocarbon contents are enhanced upon nitrogen deprivation in a subisolate of *Botryococcus* sp.

N-replete	Individual measurements			Average	% CDW^a^
CDW (g/L)	1.61	1.58	1.63	1.61 ± 0.03	
TL (g/L)	0.59	0.6	0.56	0.58 ± 0.02	36.0
HC (g/L)	0.30	0.36	0.28	0.31 ± 0.04	18.3

N-depleted	Individual measurements			Average	% CDW
CDW (g/L)	1.67	1.67	1.70	1.68 ± 0.02	
TL (g/L)	1.25	1.29	1.23	1.25 ± 0.03	74.4
HC (g/L)	0.87	0.84	0.92	0.87 ± 0.04	51.8

^a^CDW for cell dry weight.

To investigate whether ND treatment would enhance the lipid and/or hydrocarbon content, the log-phase cells were subjected to ND by replacing BB-N medium (or nitrogen-less BB medium) without altering the cell density. Three days after ND, cell density was slightly increased by 4 % of CDW (i.e., from 1.61 to 1.68 g/L). Nile red is a fluorescent hydrophobic probe that is commonly used for detection of cellular neutral lipid and hydrocarbon deposits [31]. Fluorescence microscopic analysis of Nile red-stained subisolate cells showed that many tiny lipid deposits were visible in cells prior to ND, which were likely composed of triacylglycerols or hydrocarbons or both (Fig. 1b). On the other hand, these tiny deposits were aggregated to form much large lipid droplets upon ND. This result suggests that contents of lipids (i.e., triacylglycerols or hydrocarbons or both) are enhanced by ND in this subisolate.

*Botryococcus* cells tended to form bundles, which made it unsuitable for FACS analysis to determine lipid contents in individual cells. Hence, we applied gravimetric method to estimate total lipid and hydrocarbon contents in cells prior to and after ND (see “Methods”). To this end, total lipid (i.e., extracted by methanol/chloroform) and hydrocarbon (i.e., extracted by hexane) contents were found to increase by 2.2-fold and 2.8-fold, respectively (see Table 2). This result indicated that the lipid and hydrocarbon contents in the subisolate were highly enhanced upon ND.

The methanol/chloroform extract or total lipid was subsequently subjected to thin-layer chromatographic analysis. Our results indicated that levels of triacylglyceride (TAG), diacylglyceride (DAG), free fatty acid (FFA), but not monoacylglyceride (MAG) were increased in the subisolate upon ND (Fig. 1c). These results indicated that the moderately growing subisolate’s neutral lipid accumulation was enhanced by ND.

### The subisolate belongs to the group of race A *B. braunii*

**Fig. 2:**
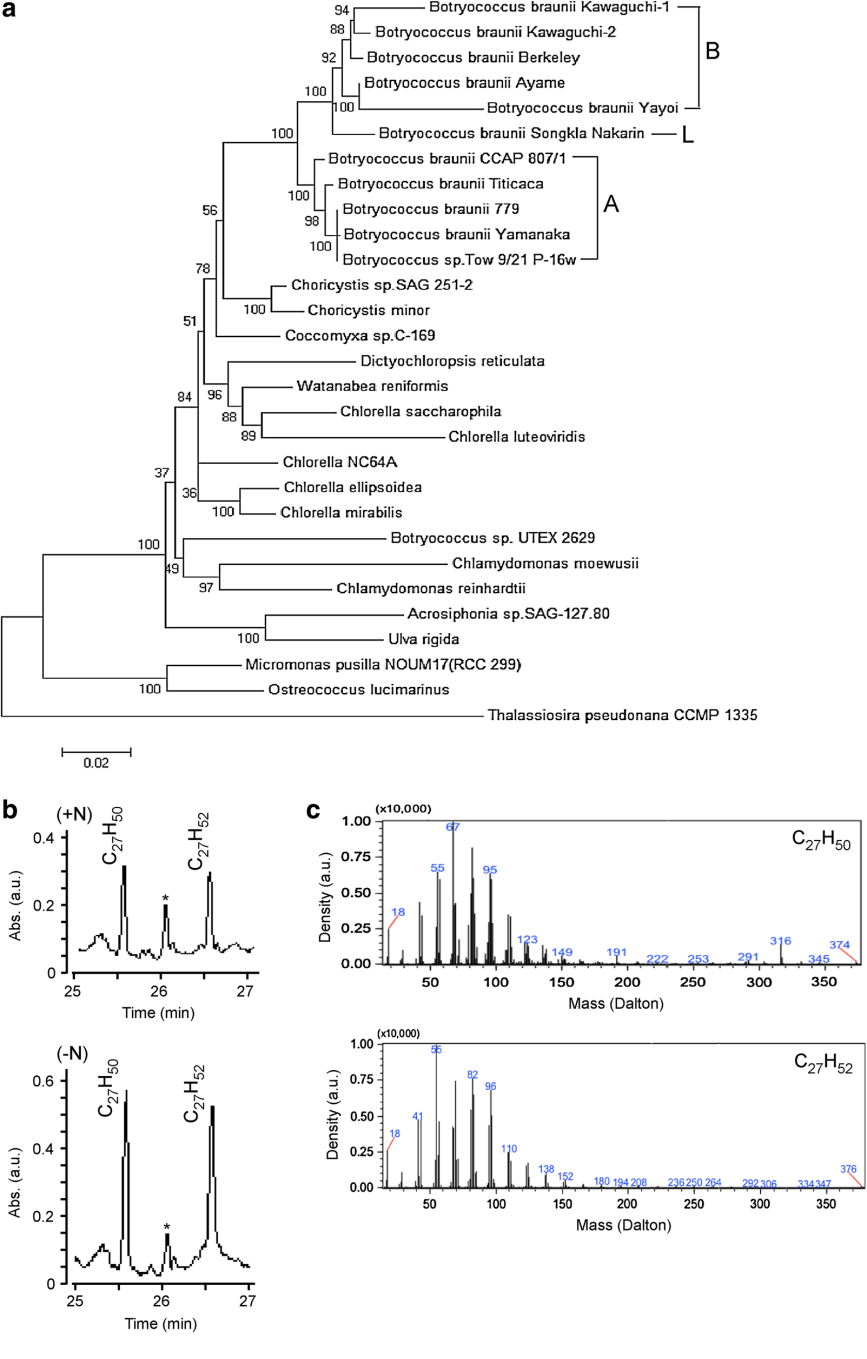
The subisolate belongs to the group of *Botryococcus braunii* race A. **a** Phylogenetic analysis of the subisolate with other *Botryococcus* spp. based on the 18S rDNA sequence. Groups of *Botryococcus* spp. race A, B, L are indicated. The subisolate of CCALA 779 in the phylogenetic tree is indicated. **b** GC spectrum of hydrocarbons extracted from cells of the subisolate prior to and after ND. X- and Y-axis indicate time in minute (min) and absorbance (abs) in arbitrary unit (a.u.). Compositions of hydrocarbon are indicated. The *asterisk* (*) indicates an unknown chemical species. **c** Mass spectra of the heptacosdiene C_27_H_52_ and heptacostriene C_27_H_50_. X- and Y-axis indicate mass in Dalton and density in arbitrary unit (a.u.), respectively.

To investigate the phylogenic placement of this subisolate, we performed the 18S rDNA sequences-based phylogenetic analysis. For this reason, the 18S rDNA sequences were amplified using PCR with sequence-specific primers [32] (see “Methods”). Our result indicated that the subisolate was mostly related to the group of A race *B. braunii* spp. (Fig. 2a). It is known that A race *B. braunii* accumulates mainly the *n*-alkadiene and alkatriene [13], unlike B and L races of *B. braunii* that accumulate largely botryococcenes (or triterpenoids) and lycopadiene (or tetraterpenoids), respectively [14, 15].

To investigate whether the subisolate contained *n*-alkadiene and triene, the hexane extract was subjected to GC–MS analysis. To this end, heptacosdiene (C_27_H_52_) and heptacostriene (C_27_H_50_) were found to be the predominant composition in the hydrocarbon extract (Fig. 2b, c), supporting the 18S rDNA sequence-based phylogenetic analysis. Hence, we designated this subisolate as A race *B. braunii* 779. We found that the contents of total hydrocarbons (see Table 2), possibly including heptacosdiene and heptacostriene were increased upon ND in *B. braunii* 779.

### The de novo-assembled transcriptome of *B. braunii* 779 appears to mostly resemble that of *Coccomyxa subellipsoidea*

To investigate the transcriptomic change upon ND, we first wanted to assemble the *B. braunii* 779 transcriptome. For this reason, *B. braunii* 779 log-phase growing cells prior to and 3 days after ND were collected for total RNA extraction (see “Methods”). The resulting total RNA samples were individually subjected to sequencing analysis using the Illumina HiSEQ 2000 platform (BGI). Over 27 million good reads with 90 nt in length were obtained from each sample. All 55 million reads were pooled for de novo assembly of transcriptome using Trinity software [33] (see “Methods”).

**Fig. 3:**
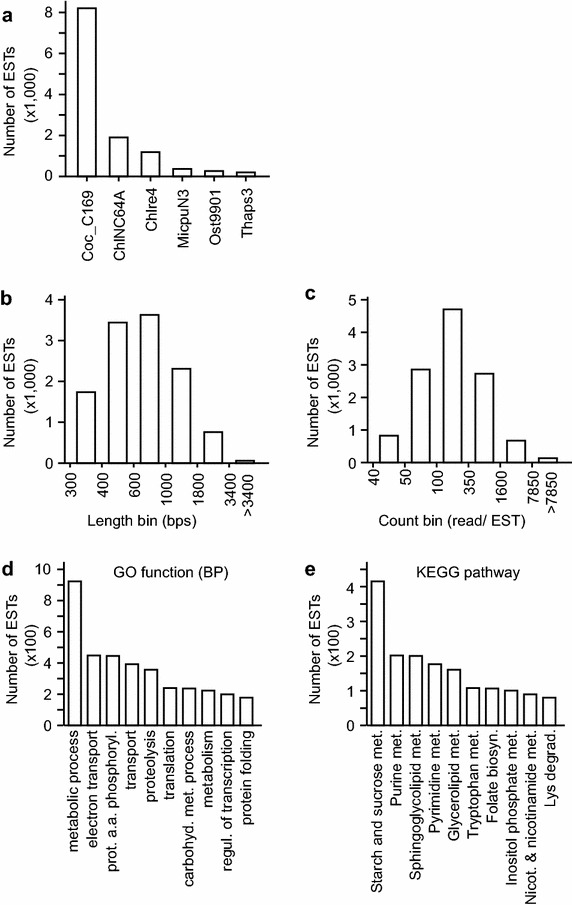
Characteristics of the de novo-assembled *B. braunii* 779 transcriptome. **a** Distribution of number of best-hits derived from the genome of various algal species. X- and Y-axis indicate the genome of algal species used and the number of ESTs in *B. braunii* 779. **b** Length and **c** count distribution of the 21,292 annotated ESTs in *B. braunii* 779. X- and Y-axis indicate the EST length (*left panel*) and count (*right panel*) and number of ESTs, respectively. **d** Ten GO biological process categories associated with the most number of ESTs in the transcriptome of *B. braunii* 799. **e** Ten KEGG pathways associated with the most number of ESTs in the transcriptome of *B. braunii* 799.

We obtained 61,220 non-redundant ESTs based on the cutoff of identity <90 % (i.e., based on CD-Hit analysis), EST length >300 bps, and read count per EST >40. Annotation of the non-redundant ESTs was performed based on the best-hit proteins/genes generated by sequence homology comparison using the Basic Local Alignment Search Tool BLASTX suite (http://blast.ncbi.nlm.nih.gov) against the “best” proteins/genes in the comprehensively annotated genomes of 5 green microalgae *Coccomyxa subellipsoidea* [34], *Chlorella variabilis* [35], *Chlamydomonas reinhardtii* [36], *Micromonas pusilla* [37], and *Ostreococcus lucimarinus* [38] and a brown alga *Thalassiosira pseudonana* [39] with a cutoff expectation value of 1E−07 (see “Methods”). To this end, a total of 12,292 (20.1 %) of the non-redundant ESTs in *B. braunii* 779 were shown to have best-hits (i.e., e value <1.0E−07) (for a complete list of annotated ESTs, see Additional file 1: Table S1). We found that majority (~70 %) of the best-hits in *B. braunii* 779 were derived from *C. subellipsoidea*, suggesting that the transcriptome of *B. braunii* 779 is most closely related to that of *C. subellipsoidea* (Fig. 3a). This observation was supported by the result of the 18S rDNA sequence-based phylogenetic analysis (see Fig. 2a).

The 12,292 annotated ESTs in the transcriptome of *B. braunii* 779 were subjected to functional analysis in this study. The length of these ESTs was ranged from 301 bps to 8,268 bps with the median length of 663 bps (Fig. 3b). The read count per EST was ranged from 42 to 182,192 with the median of 174. Of the 12,292 ESTs, 8,888 were found to be associated with at least one gene-ontology (GO) function. A total of 539 GO functions in Biological Process, 171 functions in Cellular Component, and 1,549 functions in Molecular Function associated to the *B. braunii* 779 transcriptome (for a complete list of EST-associated GO functions, see Additional file 2: Table S2). Ten GO biological processes associated with the most number of ESTs are listed in Fig. 3d (for top 10 CC and MF, see Additional file 3: Figure S1).

Subsequently we found that 3,386 out of the 12,292 ESTs were associated with at least one KEGG ortholog. The metabolic pathways associated with the most number of ESTs are shown in Fig. 3e (for a complete list of EST-associated pathways, see Additional file 4: Table S3). Richness of the GO and KO (KEGG ortholog) annotation of the transcriptome allowed performing pathway-based transcriptional analysis (see “Methods”).

### Majority of the ESTs in the previously reported A race, but not B race transcriptome of *B. braunii* are found in the transcriptome of *B. braunii* 779

**Fig. 4:**
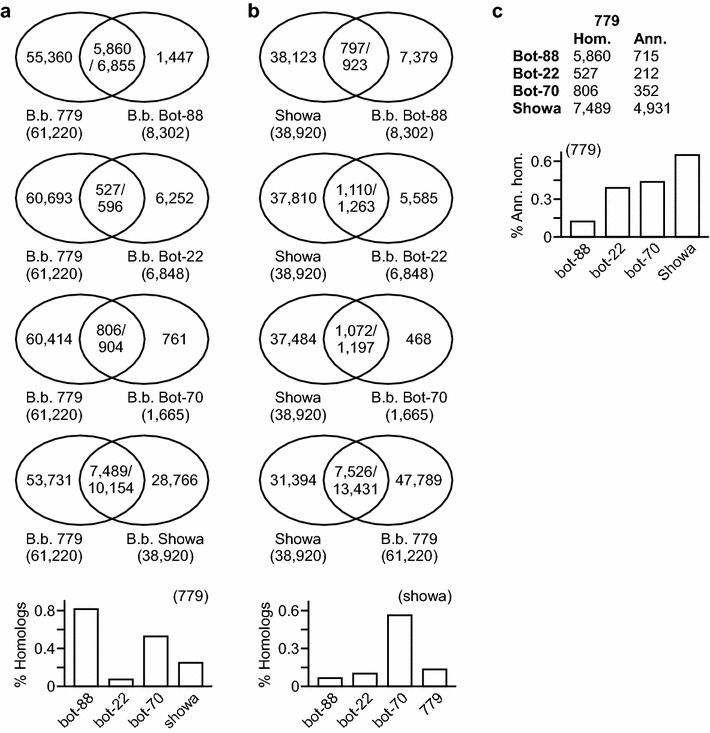
Highest level of similarities is found between transcriptomes of the same race *B. braunii* strains. **a** Highest level of similarity is observed between transcriptomes of *B. braunii* Bot-88 and 779. Venn diagrams show the homologous ESTs between the query transcriptome (e.g., Bot-88, Bot-22, Bot-70, or Showa) and the subject transcriptome 779. Percent of homologous ESTs in query transcriptomes is shown at the *bottom*. **b** Highest level of similarity is found between the query transcriptomes of *B. braunii* Showa and Bot-70. Percent of homologous ESTs in query transcriptomes is shown at the *bottom*. **c** Lowest percentage of the known sequences is found in the homologous ESTs between *B. braunii* 779 and Bot-88. Number of all homologous (Hom.) ESTs and annotated (Ann.) ESTs between *B. braunii* 779 and other transcriptomes are listed on the upper panel. A *bar plot* at *bottom* shows the percent of annotated ESTs in various sets of homologous ESTs in *upper panel.*

To compare the similarity of the *B. braunii* 779 transcriptome with the previously reported transcriptomes of A race *B. braunii* Bot-88 and B race *B. braunii* Showas, Bot-70, and Bot-22, we first processed the datasets as did for the *B. braunii* 779 by removing any ESTs/cDNAs/contigs (for consistency, we use ESTs) whose length was less than 300nts and CD-Hit identity was 90 % or greater in the transcriptomes of *B. braunii* Bot-88, Showa, Bot-70, and Bot-22 [21, 23–25, 35]. To this end, we obtained sets of 8,302, 38,920, 1,665, and 6,848 ESTs in transcriptomes of *B. braunii* Bot-88, Showa, Bot-70, and Bot-22, respectively. The processed transcriptomes were subjected to BLASTN analysis (http://blast.ncib.nlm.nih.gov) against the transcriptome of *B. braunii* 779. Based on a cutoff of e value 1E−05, we found that, compared to others, the A race *B. braunii* Bot-88 transcriptome shared the highest percentage of ESTs to that of *B. braunii* 779 (Fig. 4a, for a complete list of homologous ESTs, see Additional file 5: Tables S4–S7).

Among the 3 published B race *B. braunii* transcriptomes, Showa had the most completed transcriptome [21, 23, 25]. Hence, the Showa transcriptome was used as reference for B race *B. braunii* transcriptome. Sequences of *B. braunii* Bot-88, Bot-22, Bot-70, and 779 transcriptomes were subjected to homology analysis using BLASTN against that of *B. braunii* Showa. With a cutoff of e value 1E−05, we found that, among others, the B race *B. braunii* Bot-70 shared the highest percentage of ESTs to that of *B. braunii* Showa (Fig. 4b, for a complete list of homologous ESTs, see Additional file 6: Tables S8–S11). It was noted that majority (i.e., ~75 %) of transcriptomes between *B. braunii* A race 779 and B race Showa were found to be different, implying that A and B races belong to different sub-species of *B. braunii*.

Of the 5,860 *B. braunii* 779 ESTs that shared homolog to A race *B. braunii* Bot-88, we noted that only 715 (or 12.2 %) had best-hit in the “best” proteins of the 6 comprehensively annotated genomes (Fig. 4c). On the other hand, 40–60 % of the *B. braunii* 779 sheared homologous ESTs to the B races appeared to have functional annotations. This result implied that many ESTs in *B. braunii* 779 or Bot-88 without a best-hit in the 6 selected genomes might represent novel genes specific to A race *B. braunii* species.

### ESTs associated with energy metabolisms are abundantly expressed in the log-phase *B. braunii* 779 cells

Individual EST levels were normalized to FPKM (i.e., Fragments Per Kilobase of EST per Million fragments mapped) and averaged from two biological repeated experiments for functional analysis of the transcriptome. Based on association with GO functions, we found that, of the top 30 most abundant ESTs ranked by level, 21 were associated with photosynthesis function (or light harvesting, GO:0009765) that was greatly enriched (i.e., 239-fold increase, *p* value <1E−26). Besides, glycolysis (i.e., GO:0006096) and translation (i.e., GO:0006412) were also enriched by 20-fold and sixfold (i.e., *p* value = 4.3E−03 and 8.7E−03), respectively (Table 3).

**Table 3:** List of the top 30 most abundant ESTs in log-phase *B. braunii* 779 cells

Rk^a^	EST_ID^b^	BH_ID^c^	FPKM^d^	Description^e^
1	c72167_g1_i4	ChlNC64A_1|59790	12,471.445	Photosynthesis, light harvesting
2	c72167_g1_i8	Coc_C169_1|58975	10,031.55	Photosynthesis, light harvesting
3	c72102_g4_i5	Coc_C169_1|27246	7,119.695	Photosynthesis, light harvesting
4	c72167_g1_i2	Coc_C169_1|58975	6,831.27	Photosynthesis, light harvesting
5	c72167_g1_i6	Coc_C169_1|27246	6,731.515	Photosynthesis, light harvesting
6	c72102_g4_i4	Coc_C169_1|58975	4,540.985	Photosynthesis, light harvesting
7	c72167_g1_i7	Coc_C169_1|27246	3,366.845	Photosynthesis, light harvesting
8	c72102_g4_i1	Coc_C169_1|58975	2,639.815	Photosynthesis, light harvesting
9	c68174_g2_i1	Coc_C169_1|30477	2,360.575	60S ribosomal protein L23
10	c68658_g2_i1	Coc_C169_1|35121	2,110.295	Photosynthesis, light harvesting
11	c57826_g1_i1	Coc_C169_1|44136	1,790.37	Photosynthesis, light harvesting
12	c58876_g1_i1	Coc_C169_1|28488	1,713.8	Photosynthesis, light harvesting
13	c54674_g1_i1	Coc_C169_1|64185	1,710.555	Photosynthesis, light harvesting
14	c72326_g1_i2	Coc_C169_1|67011	1,573.705	Photosynthesis, light harvesting
15	c72522_g1_i1	Coc_C169_1|34109	1,538.055	Fructose-biphosphate aldolase
16	c70416_g1_i1	Coc_C169_1|27513	1,432.875	Translation elongation factor EF-1
17	c65010_g1_i1	Coc_C169_1|19247	1,314.1	Photosynthesis, light harvesting
18	c62135_g1_i1	Chlre4|184775	1,184.59	Photosynthesis, light harvesting
19	c72592_g1_i1	Coc_C169_1|35576	1,170.825	Enolase
20	c71666_g1_i1	Chlre4|140045	1,082.22	Ubiquitin and ubiquitin-like proteins
21	c58350_g1_i1	Coc_C169_1|28397	1,073.435	60S ribosomal protein L35
22	c72102_g5_i1	Coc_C169_1|28488	977.63	Photosynthesis, light harvesting
23	c70207_g1_i2	ChlNC64A_1|59626	859.85	Photosynthesis, light harvesting
24	c70122_g2_i2	Coc_C169_1|53355	839.985	Glyceraldehyde 3-phosphate dehydrogenase
25	c69841_g1_i1	Coc_C169_1|25284	823.07	Photosynthesis, light harvesting
26	c69841_g1_i3	Coc_C169_1|25284	818.15	Photosynthesis, light harvesting
27	c57090_g1_i1	Coc_C169_1|26429	790.785	60S ribosomal protein L44
28	c68918_g1_i1	Coc_C169_1|37969	772.455	Photosynthesis, light harvesting
29	c61023_g1_i1	Coc_C169_1|34169	689.075	60S ribosomal protein L30
30	c68918_g1_i2	Coc_C169_1|37969	676.53	Photosynthesis, light harvesting

^a^Rk stands for rank based on average level of FPKM.

^b^EST_ID for ID of the *B. braunii* 779 ESTs.

^c^BH_ID for EST’s best-hit consistent of species and protein ID.

^d^FPKM for average level of individual ESTs.

^e^Description is based on GO annotations.

**Fig. 5:**
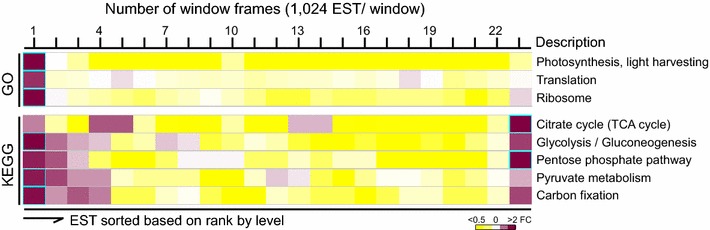
Subsets of ESTs in a number of GO biological processes and KEGG metabolic pathways are most abundantly transcribed in log-phase *B. braunii* 779 cells. Distribution of ESTs associated with 3 GO functions and 5 pathways based on ranks by level is shown. The ratio between EST density in a window and background is indicated by color (see *color* *key* at the *bottom*). Windows with significantly enriched ESTs associated with individual pathways are *boxed* (i.e., fold change >2, p value <0.05 after Bonferroni correction). *Number of windows* is indicated at the *top*.

We assumed that a subset of ESTs in a pathway displaying a similar expression level (i.e., based on ranks) or expression coherence was likely to be co-regulated by a common transcriptional regulatory network. To search for potentially co-regulated transcriptional networks, we performed the sliding window analysis using a window size of 1024 ESTs and a moving step of 512 ESTs along the sorted ESTs based on rank by level (see “Methods”). According to 44 GO biological processes and 59 KEGG metabolic pathways (i.e., number of process- or pathway-associated EST >30), we found that 2 biological processes (i.e., photosynthesis and translation) and 5 metabolic pathways (i.e., TCA cycle, glycolysis/gluconeogenesis, pentose phosphate pathway, pyruvate metabolism, and carbon fixation) whose ESTs were enriched in 1 of the 23 windows alone the sorted ESTs based on rank by level (Fig. 5). Photosynthesis and translation processes and glycolysis/gluconeogenesis, pyruvate metabolism and carbon fixation pathways were enriched in the window containing the highest ranked ESTs or in window 1 (i.e., fold change >2, *p* value <0.05 after Bonferroni correction [40, 41]). Similar to translation, ribosome component-encoding ESTs were also enriched in window 1 (i.e., fold change >2, p value <0.05 after Bonferroni correction). On the other hand, ESTs associated with the TCA cycle and pentose phosphate pathway were enriched the window containing the least abundant ESTs or in window 23 (i.e., fold change >2, p value <0.05 after Bonferroni correction). Photosynthesis (or light harvesting) and carbon fixation were essential for cell growth under photoautotrophic conditions. High-level expression of these functions in log-phase *B. braunii* cells indicated that the slow growth was not a result of inefficient energy metabolisms, rather than its devoting major portion of its energy flow to the lipid and hydrocarbon synthesis.

### ESTs encoding photosynthesis and ribosome functions are down-regulated upon ND in *B. braunii* 779

**Fig. 6:**
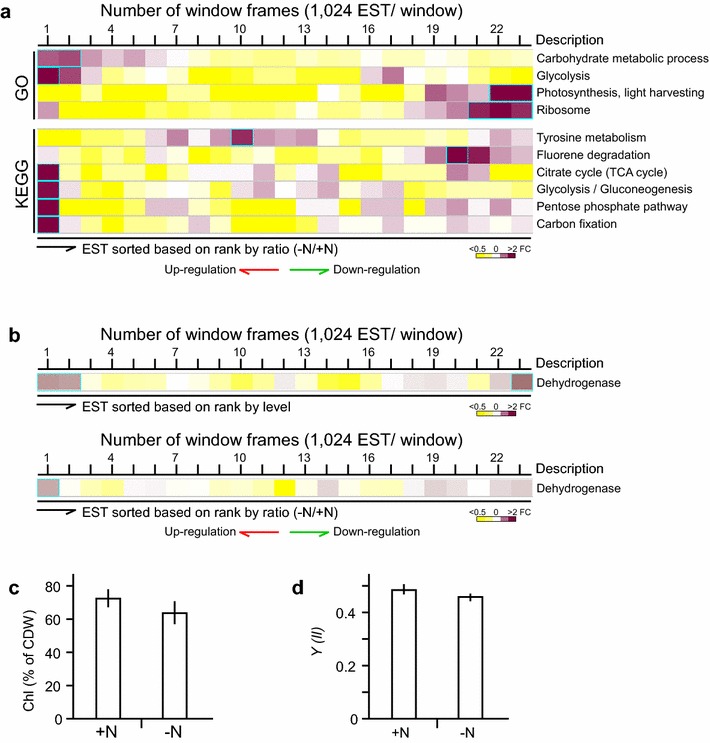
Subsets of ESTs in a number of processes and pathways are most up-regulated and down-regulated upon ND in *B. braunii* 779 cells. **a** Distribution of ESTs associated with 4 GO functions and 6 KEGG pathways based on ranks by ratio. **b** Distribution of ESTs associated with unspecific dehydrogenase based on ranks by level (*upper panel*) and ratio (*lower panel*). The display is identical to Fig. 5. **c** Levels of chlorophyll content. The *bar* *plot* shows the average level of chlorophyll contents in cells prior to (+N) and after (−N) ND. *Error bar* is derived from triplicates. **d** Efficiency of quantum yield of photosystem II. The *bar plot* shows the average level of efficiency of quantum yield (*Y*) of photosystem II in cells prior (+N) to and after (−N) ND.

To investigate changes of transcriptional activities upon ND, levels of individual ESTs after ND were compared with those prior to ND. We assumed that if a subset of ESTs associated with a pathway was enriched within a window of 1024 ESTs ranked by ratio (i.e., fold change >2, p value <0.05 after Bonferroni correction), the subset of ESTs was potentially co-regulated by a common transcription regulatory network in response to ND. Using the sliding window approach along the sorted ESTs based on rank by ratio, we found that subsets of ESTs associated with 3 GO biological processes (i.e., carbohydrate metabolism, glycolysis, and photosynthesis) and 6 KEGG metabolic pathways (i.e., tyrosine metabolism, fluorene degradation, TCA cycle, glycolysis/gluconeogenesis, pentose phosphate pathway, carbon fixation) plus ribosome subunit were enriched in one of the 23 sliding windows (Fig. 6a).

We found that subsets of ESTs associated with carbohydrate metabolism and glycolysis processes or TCA cycle, glycolysis/gluconeogenesis, pentose phosphate pathway, and carbon fixation metabolic pathways were enriched in the top most up-regulated window (see Fig. 6a). This was puzzling because cell growth would be arrested upon ND. However, it was proposed that ND induced oxidative stress that in turn triggered reconfiguration of metabolic flux to provide the reducing power for the main cellular redox systems [42, 43]. Hence, we hypothesized that the up-regulation of citrate cycle, glycolysis/gluconeogenesis, pentose phosphate pathway, and carbon fixation metabolisms was a result of reconfiguration of carbohydrate metabolisms for production of reducing power upon ND.

To test this possibility, we investigated all ESTs that were associated with unspecific dehydrogenase activity based on annotation of “dehydrogenase”. We found that unspecific dehydrogenase ESTs were enriched in 20 metabolisms (i.e., fold change >2, p value <0.05) including citrate cycle, glycolysis/gluconeogenesis, pentose phosphate pathway, and carbon fixation metabolisms. ESTs associated with unspecific dehydrogenase were found to be up-regulated upon ND (i.e., fold change >1.5, p value <0.05 after Bonferroni correction) (Fig. 6b). This result implied that up-regulation of the unspecific dehydrogenase-enriched metabolisms such as citrate cycle, glycolysis/gluconeogenesis, pentose phosphate pathway, and carbon fixation is likely a result of reconfiguration of metabolic flux to provide the reducing power for the main cellular redox systems.

We found that ESTs encoding photosynthesis (or light harvesting) and ribosome functions were enriched in the most down-regulated windows (i.e., ranked at bottom by ratio) (see Fig. 6a). It was proposed that excess proteins involved in light harvesting and protein synthesis were used as nitrogen-rich molecules to recycle nitrogen during ND [22]. To test this possibility, we measured chlorophyll content and photosynthetic yield in cells prior to and after ND. Although transcription level of ESTs associated with light harvesting and ribosome function was dramatically down-regulated, the level of chlorophyll and photosynthetic yield was only marginally affected (Fig. 6c, d). This result implied that excess transcripts encoding photosynthesis function and ribosomes were recycled for nitrogen hardly affecting the photosynthetic efficiency upon ND in *B. braunii* 779.

### Up- and down-regulated ESTs induced by ND appear to be low- and high-abundant ESTs in log-phase *B. braunii* 779 cells, respectively

**Fig. 7:**
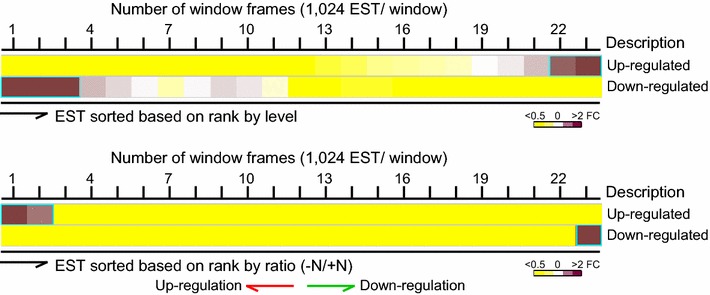
Significantly up-regulated and down-regulated ESTs upon ND appear to be low-abundant and high-abundant ESTs in log-phase cells (prior to ND), respectively. Distribution of the differentially transcribed ESTs (i.e., fold change >2, p value <0.05) based on the level-ranked (*upper panel*) and ratio-ranked (*lower panel*) moving window analysis.

Based on the duplicate, read count per EST was estimated by RSEM [44] and subsequently the differential expression level was determined using EdgeR [45]. To this end, we obtained 593 and 116 differentially up- and down-regulated ESTs in response to ND in *B. braunii* 779, respectively, based on a cutoff of fold change >2 and p value <0.05 (see “Methods”, for a complete list of differentially transcribed ESTs, see Additional file 7: Table S12). Lists of the top 25 up-regulated and down-regulated ESTs are shown in Tables 4 and 5. We found that many up-regulated ESTs upon ND (i.e., fold change >2, p value <0.05) appeared to be the least abundant ESTs prior to ND (Fig. 7). Though nitrogen metabolic pathway was not up-regulated upon ND, we found that 7 ESTs (i.e., 1 ammonia permease, 2 glutamine synthases, and 4 glutamate synthases) out of the top 25 most up-regulated ones were involved in nitrogen metabolism (see Table 4). On the other hand, many down-regulated ESTs upon ND tended to be relatively highly abundant in cells prior to ND (see Fig. 7).

**Table 4:** List of the top 25 most up-regulated ESTs upon ND in *B. braunii* 779

Rk^a^	EST_ID^b^	BH_ID^c^	logFC	p value	Description^d^
1	c39922_g1_i1	Ost9901_3|32601	12.06	1.2E−13	Ammonia permease
2	c63389_g1_i3	Coc_C169_1|23517	10.56	4.1E−09	Glutamine synthetase
3	c9966_g1_i1	Chlre4|188119	10.36	3.6E−09	Oxidoreductase
4	c27174_g1_i1	Coc_C169_1|52379	9.57	8.9E–09	Glycogen phosphorylase
5	c60040_g1_i1	Coc_C169_1|32937	9.49	1.3E−08	Pyruvate kinase
6	c90800_g1_i1	ChlNC64A_1|143431	9.48	2.3E−09	Glutamine synthetase
7	c71872_g1_i2	Ost9901_3|50765	9.19	2.8E−10	Sulfite reductase
8	c52649_g1_i1	Coc_C169_1|52379	8.65	1.0E−07	Glycogen phosphorylase
9	c81537_g1_i1	Chlre4|206116	8.04	4.1E−08	Unknown
10	c67134_g2_i2	MicpuN3|63658	7.94	1.3E−06	Glutamate synthase
11	c78877_g1_i1	Chlre4|140452	7.77	5.1E−07	Unknown
12	c49437_g1_i2	MicpuN3|97997	7.67	1.2E−06	Unknown
13	c9210_g1_i2	Chlre4|132210	7.66	1.7E−07	3-phosphoglycerate kinase
14	c70762_g1_i7	Chlre4|140487	7.65	1.8E−07	Glutamate synthase
15	c59003_g1_i1	MicpuN3|63658	7.45	9.2E−06	Glutamate synthase
16	c19522_g1_i1	Thaps3|21748	7.24	6.4E−07	Fructose 1,6-bisphosphate aldolase
17	c20385_g1_i1	Ost9901_3|30705	7.14	9.0E−07	Cohesin subunit
18	c27189_g1_i1	Thaps3|27187	6.88	2.4E−07	Transaldolase
19	c24873_g1_i2	MicpuN3|89262	6.88	1.3E−06	FOG: RCC1 domain
20	c51175_g1_i1	Coc_C169_1|29458	6.87	3.8E−06	Citrate synthase
21	c47749_g1_i1	Coc_C169_1|9065	6.87	1.0E−07	Transcription factors
22	c61180_g1_i2	Chlre4|205746	6.79	7.9E−06	Glutamate synthase
23	c2096_g1_i1	MicpuN3|82943	6.76	4.0E−07	RNA Helicase
24	c36160_g1_i3	Thaps3|262283	6.74	5.6E−07	Acyl-CoA synthetase
25	c27223_g1_i2	Thaps3|269844	6.71	4.0E−07	UDP-glucuronosyl transferase

^a^Rk stands for rank (i.e., by ratio).

^b^EST_ID for *B. braunii* ESTs.

^c^BH_ID for ID of the best-hit.

^d^Description is based on the KOG annotation associated with the 6 completed algal genomes in JGI (see text).

**Table 5:** List of the top 25 most down-regulated ESTs upon ND in *B. braunii* 779

Rk^a^	EST_ID^b^	BH_ID^c^	logFC	p value	Description^d^
1	c73365_g3_i6	Coc_C169_1|45719	−4.55	6.4E−03	Unknown
2	c54903_g1_i1	Coc_C169_1|45718	−4.40	2.5E−03	Tyrosine kinase
3	c72890_g1_i2	ChlNC64A_1|144962	−4.20	1.8E−03	Prolylcarboxypeptidase
4	c69499_g1_i5	Chlre4|149722	−4.18	4.5E−03	Unknown
5	c66708_g1_i4	Coc_C169_1|65911	−3.98	2.8E−04	Collagens
6	c72757_g1_i1	Coc_C169_1|17756	−3.54	1.5E−03	Isocitrate lyase
7	c66635_g1_i1	ChlNC64A_1|139124	−3.46	2.8E−03	Nuclear receptor coregulator
8	c32919_g1_i2	Coc_C169_1|18195	−3.44	2.1E−04	Aspartate aminotransferase
9	c71206_g1_i2	Coc_C169_1|62318	−3.36	2.0E−02	UDP-glucuronosyl transferase
10	c68672_g1_i4	Coc_C169_1|65911	−3.31	9.4E−04	Collagens
11	c55715_g1_i1	Coc_C169_1|68180	−3.27	6.9E−04	Ca^2+^-dependent protein kinase
12	c71767_g1_i2	Chlre4|104719	−3.23	5.1E−03	ATP-dependent RNA helicase
13	c69499_g1_i4	Chlre4|149722	−3.16	1.7E−02	Unknown
14	c73427_g1_i1	Coc_C169_1|35165	−3.01	1.0E−02	O-linked GlcNAc transferase
15	c66185_g1_i1	Coc_C169_1|33465	−3.01	1.6E−02	Unknown
16	c67100_g1_i1	Chlre4|130199	−3.01	2.2E−02	Acetylglutamate kinase
17	c71718_g1_i18	Coc_C169_1|68054	−3.01	4.4E−03	Ca^2+^-permeable cation channel
18	c70935_g1_i8	Coc_C169_1|13500	−3.00	4.4E−03	Unknown
19	c51991_g1_i1	Coc_C169_1|63373	−2.99	1.2E−02	Diacylglycerol acyltransferase
20	c74265_g5_i3	Coc_C169_1|30369	−2.94	3.9E−03	Malate synthase
21	c73301_g5_i1	Coc_C169_1|17144	−2.94	1.5E−02	Serine/threonine protein kinase
22	c71077_g1_i3	ChlNC64A_1|59702	−2.94	8.1E−03	Unknown
23	c73505_g1_i1	Coc_C169_1|41290	−2.92	2.0E−03	Unknown
24	c74003_g1_i7	Coc_C169_1|35165	−2.89	6.3E−03	O-linked GlcNAc transferase
25	c62188_g1_i1	Coc_C169_1|11065	−2.89	9.6E−03	Sensory transduction histidine kinase

^a^Rk stands for rank (i.e., by inverse ratio).

^b^EST_ID for *B. braunii* ESTs.

^c^BH_ID for ID of the best-hit.

^d^Description is based on the KOG annotation associated with the 6 completed algal genomes in JGI (see text).

### Transcriptional alteration of ESTs involved in alkadiene/triene-related biosynthesis of VLCFA upon ND in *B. braunii* 779

A subset of 55 enzymes that were potentially involved in the biosynthesis of VLCFA was proposed by Baba et al., out of which they detected 19 enzymes in the A race *B. braunii* Bot-88 [24]. Based on association with these EC numbers, we detected 20 enzymes in the *B. braunii* 779 transcriptome, of which, 11 were those previously found in *B. braunii* Bot-88 [24], suggesting that A race *B. braunii* strains share many VLCFA biosynthetic enzymes (i.e., p value = 0.0137).

**Fig. 8:**
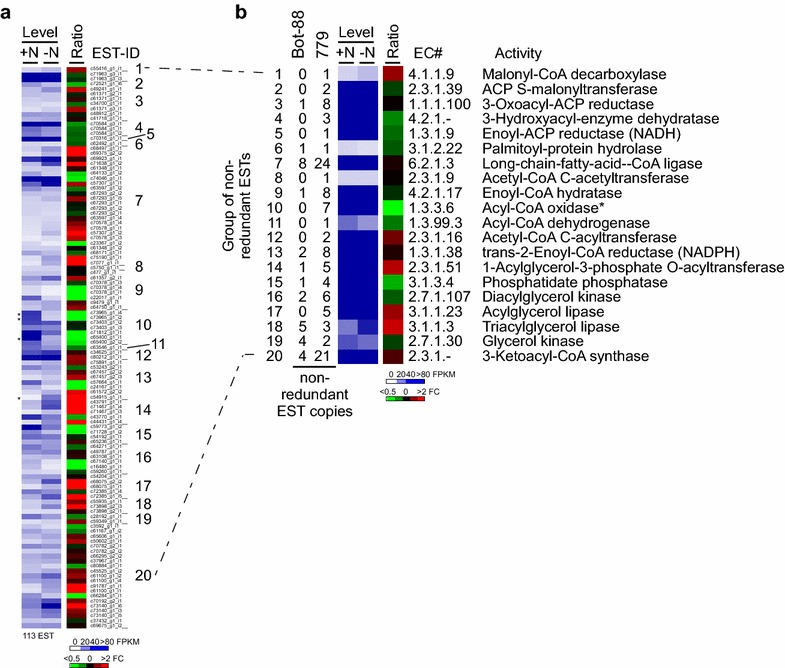
Change of transcriptional levels of ESTs involved in VLCFA biosynthesis upon ND. +N and −N indicate prior to and after ND, respectively. Individual enzymes are numbered and whose EC# is indicated. Many enzymes are encoded by various numbers of non-redundant ESTs in *B. braunii* (779) or (Bot-88). **a** Level and ratio of individual EST involved in VLCFA biosynthesis in cells prior to (+N) and after (−N) ND are shown. Differentially transcribed ESTs upon ND are marked by *asterisks* on the *left*. **b** Level and ratio of group ESTs (i.e., sum of individual levels of ESTs encode the same enzyme) involved in VLCFA biosynthesis are shown. Activity whose summative level is greatly changed (i.e., fold change >2) is indicated by an *asterisk*.

We found that each enzyme was encoded by various copies of the non-redundant ESTs (Fig. 8a). Levels of individual copies of non-redundant ESTs ranged from 1.9 to 110 FPKM in cells prior to and from 1.9 to 80 FPKM after ND based on the average of two repeats. There were 1 significantly up-regulated and 3 down-regulated ESTs (i.e., fold change >2, p value <0.05). After summation of levels of all ESTs associated with the same enzyme, we found that the summative level of all enzymes tested was not greatly altered (i.e., fold change <2) upon ND except for acetyl-CoA oxidase (i.e., >twofold decrease) (Fig. 8b, for summative level of individual enzymes, see Additional file 8: Table S13). This result indicated that no apparent transcriptional alteration for most enzymes (i.e., not individual ESTs) involved in VLCFA biosynthesis upon ND in *B. braunii* 779.

### Transcriptional alteration of ESTs involved in botryococcene or squalene-related biosynthesis upon ND in *B. braunii* 779

**Fig. 9:**
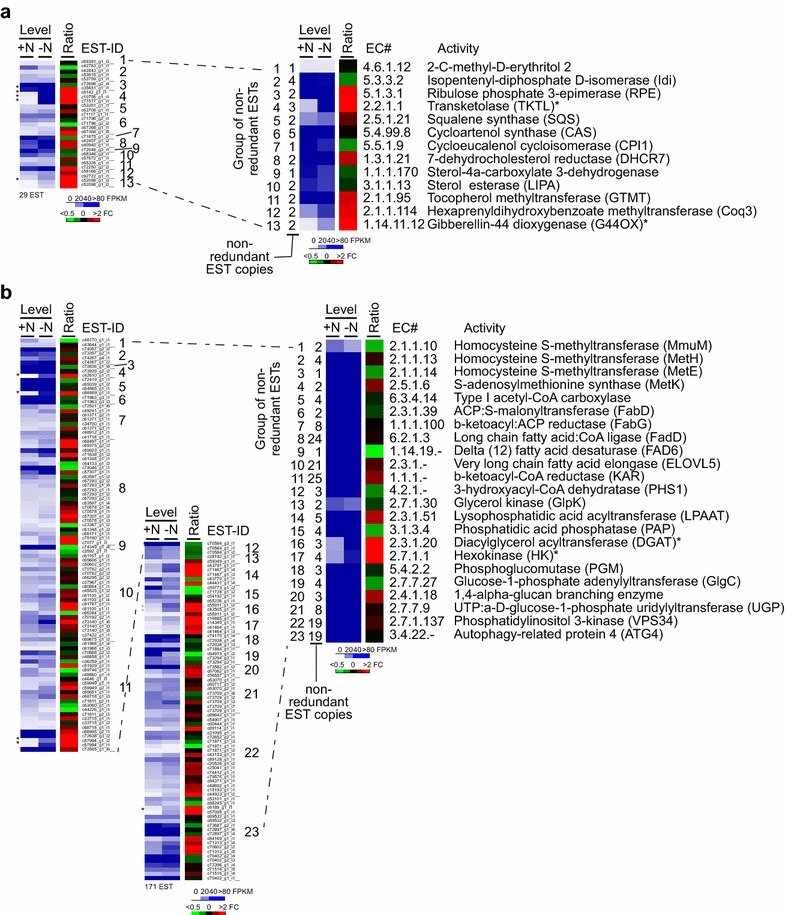
Change of transcriptional levels of ESTs involved in botryococcene or squalene biosynthesis upon ND. The display is identical to Fig. 8. **a** ESTs homologous to curated and machine-assembled **b** enzymes involved in botryococcene or squalene biosynthesis in *B. braunii* Showa [21]. The display is identical to Fig. 8.

Transcriptome of the B race *B. braunii* Showa was well assembled and comprehensively annotated [21]. To examine whether the transcription of enzymes involved in biosynthesis of botryococcene or squalene was altered upon ND in A race *B. braunii* 779, we compared the ESTs between *B. braunii* 779 and Showa based on the association with EC numbers. To this end, we found 58 and 42 enzymes related to botryococcene or squalene biosynthesis were associated with curated and machine-assembled ESTs in *B. braunii* Showa [21], respectively (see “Methods”). Of the 58 enzymes associated with curated ESTs in *B. braunii* Showa, 13 (or 22.4 %) were found to associate with a total of 29 ESTs in *B. braunii* 779 (Fig. 9a, left panel). Based on the summation of all ESTs associated with the same enzyme, we found that summative transcription level of all enzymes was not dramatically altered upon ND (i.e., fold change <2), except for TKTL and G44OX (i.e., >twofold increase) (Fig. 9a, right panel, for summative level of individual enzymes, see Additional file 9: Table S14). On the other hand, 23 out of 42 (or 54.8 %) machine-assembled enzymes in *B. braunii* Showa were found to associate with 171 ESTs in *B. braunii* 779, five of which associated with 19 or more ESTs (Fig. 9b, left panel). Similar to that of curated enzymes, transcriptional level of all enzymes was not dramatically altered upon ND (i.e., fold change <2), except for DGAT and HK (i.e., >twofold increase), based on the summative level of all ESTs associated with the same enzyme (Fig. 9b, right panel, for summative level of individual enzymes, see Additional file 10: Table S15). These results suggested that transcriptional level of most enzymes involved in botryococcene or squalene biosynthetic pathways was not significantly altered upon ND in A race *B. braunii* 779.

## Discussion

The colonial green microalga *B. braunii* is unique for its accumulation of hydrocarbons, which is applicable for biofuel production. However, slow growth has impeded its application for large-scale production of hydrocarbons. Unlike our previous report that a rapidly growing subisolate from a culture of *B. braunii* UTEX572 has turned out to be a *Botryosphaerella* (UTEX2629) containing no hydrocarbons [22], we show, in this study, that the moderately growing subisolate from a culture of *Botryococcus* sp. CCALA-779 belongs to the A race *B. braunii* based on the results of 18S rRNA sequence-based phylogenetic analysis and GC–MS analysis of hydrocarbon compositions (see Fig. 2). The lipid content of this moderately growing *B. braunii* 779 strain under phototrophic conditions can be greatly enhanced by ND, making it attractive for potential production of hydrocarbons at large scale.

In comparison with that of model green algal organisms *Chlorella variabilis* [35] and *Chlamydomonas reinhardtii* [36], growth rate *of B. braunii* is very slow (i.e., population doubling time of less than a day versus 5–7 days). In this study, we show that, according to the pathway-based transcriptomic analysis, energy metabolic ESTs are highly abundant in the log-phase cells, indicating that the slow growth rate of *B. braunii* species is not a result of inefficient energy metabolic activity in cells. It is likely that energy flow generated from photosynthesis in *B. braunii* cells is largely directed to the biosynthesis of lipid and hydrocarbon. We propose that if the direction of energy flow could be controlled in cells, the productivity would be improved by first directing the energy flow to population growth. After reaching the maximum density of cell population, energy flow would be re-directed to lipid and hydrocarbon synthesis.

It has been shown that the modes of hydrocarbon biosynthesis in A race *B. braunii* Bot-88 and B race *B. braunii* Showa, Bot-22, and Bot-70 are very different according to the 454 sequencing-based transcriptomic analyses [21, 23–25]. In this study, we performed transcriptomic analysis of the *B. braunii* 779 using the Illumina sequencing-based platform. Comparative analysis indicates that highest percentage of sequence similarities is found between transcriptomes of the same race such as A race *B. braunii* strains 779 and Bot-88 and B race strains Showa and Bot-70 (see Fig. 4). However, we find that transcriptome of Bot-22 shares little similarity to either B race *B. braunii* Showa or A race 779 strains, suggesting that it represents no A or B race transcriptome. The difference between transcriptomes of A and B races implies that they belong to different sub-species.

It is worth noting that 40–60 % of the homologous ESTs between *B. braunii* 779 and other B race strains are known sequences (or having best-hit in the 6 algal genomes used in this study). On the other hand, only 12 % of the homologous ESTs between A race *B. braunii* 779 and Bot-88 are known sequences. Majority of the homologous ESTs in A race transcriptomes are unknown sequences, suggesting that there are many novel genes in *B. braunii*. Given that many enzymes encoding VLCFA biosynthesis have not been found in A race *B. braunii*, it is possible that sequences encoding these enzymatic activities may not be currently known. Hence, we propose that *B. braunii* provides a rich source for discovery of novel genes involved in VLCFA biosynthesis.

To understand the biosynthesis of *n*-alkadiene and triene, Baba et al. have proposed a list of 55 enzymes that are involved in biosynthesis of VLCFA [24]. Nineteen out of the 55 enzymes are found in *B. braunii* Bot-88. In this study, we find 20 out of the 55 enzymes involved in the biosynthesis of the VLCFA, 13 of which are those previously found in *B. braunii* Bot-88 (i.e., p value <0.05), consistent with the notion that A race *B. braunii* strains belong to the same sub-species. Eight more enzymes involved in biosynthesis of VLCFA are found in this study. Additionally, 36 out of 100 enzymes in *B. braunii* Showa [21] involved in biosynthesis of botryococcene or squalene are found in *B. braunii* 779 (see Fig. 9), consistent with the level of similarity between the two transcriptomes (see Fig. 4).

## Conclusions

Taken together, our results show that the subisolate *B. braunii* 779 exhibits a moderate growth rate (i.e., up to 1.23 gram of CDW per liter per day in 2× BB medium) and high lipid content (i.e., up to 75 % of DCW after induction by ND), attractive for biofuel production. Comparative transcriptomic analysis between A (i.e., *B. braunii* 779 and Bot-88) and B (i.e., *B. braunii* Showa, Bot-22, and Bot-70) races indicates that difference races of *B. braunii* strains may belong to different sub-species. Many homologs of unknown sequences found in *B. braunii* 779 and Bot-88 suggest that these unknown sequences are most likely novel genes, part of which possibly encodes enzymes involved in VLCFA biosynthesis. This is consistent with the observation of half of the proposed enzymes involved in VLCFA biosynthesis has not been found in A race *B. braunii*. We propose that *B. braunii* transcriptomes provide a rich source for discovery of novel genes involved in hydrocarbon biosynthesis.

## Methods

### Algal culture manipulation

The algae culture CCALA779 was obtained from the Culture Collection of Autotrophic Organisms, Czech (ccala.butbn.cas.cz) and a subisolate *B. braunii* 779 was obtained as rapidly growing colonies on Bold’s modified Bristol (BB) [30] solid medium. The subisolate was cultivated using BB (or 2× BB) liquid medium in a 2.5-L low-form flask with shaking at 100 rpm at 25 °C under continuous illumination flux density of ~250 μmol photon m^−2^ s^−1^. Culture was shaken at 100 rpm at 25 °C (or RT) supplied with 2 % CO_2_ through bubbling. For analysis of the subisolate in response to ND, the log-phase cells in nitrogen-replete 2× BB medium (or 2BB + N) were harvested by centrifugation at 2,000 rcf at 8 °C for 5 min and resuspended in nitrogen-depleted 2× BB medium (or 2BB-N) isovolumetrically. After growing in 2BB-N medium for 3 days, cells were harvested by filtration for cell dry weight analysis and centrifugation for lipid content and transcriptomic analyses.

For growth rate analysis, cell dry weight (CDW) was determined using the gravimetric methodology. In brief, approximately 200 ml (i.e., for the first 2 time points) or 100 ml (i.e., for the time points at 4 days and after) from the cell culture was harvested by filtration using the glass fiber filter GF/A (Whatman/GE Healthcare, Kent, UK) and dried in oven at 80 °C overnight. CDW was gravimetrically determined in triplicate using the AG204 balance (Mettler-Toledo Inc., Columbus, OH, USA). Growth rate was estimated using the exponential growth formula

$$ r = \left( {\frac{{x_{{t_{2} }} }}{{x_{{t_{1} }} }}} \right)^{{1/(t_{2} - t_{1} )}} - 1 $$

where *r* is growth rate in a time interval from *t*_2_ to *t*_1_, and $x_{{t_{2} }}$ and $x_{{t_{1} }}$ is the CDW at time points *t*_2_ and *t*_1_, respectively. Here, the time interval *t*_2_ − *t*_1_ is 2 days. The growth rates at each 2-day interval from day 0 to day 8 are presented in Table 2.

### Determination of chlorophyll contents

To determine chlorophyll content, cells were collected by centrifugation and the cell pellet was resuspended in 96 % ethanol and broken in the glass-bead beater (FastPrep, MP Biomedicals, Solon, OH, USA). After incubation with ethanol for 2 h at 4 °C, cell debris was removed using centrifugation. The resulting supernatant was subjected to OD measurement at the wavelength of 645 nm and 663 nm, with the 96 % ethanol as blank. Total chlorophyll content (i.e., chl a and b) was estimated using a formula C (mg/L) = 20.2 OD_645_ + 8.05 OD_663_ [46] and was converted to percent of CDW.

### Determination of photosynthesis efficiency

To determine photosynthesis efficiency, cells were subjected to PAM-fluorescence analysis using a fluorometer (Imaging-PAM, Heinz Walz GmbH, Effeltrich, Germany) according to the manufacturer’s instruction. Briefly, the Fm′ and F were determined at the arctic light of 250 μmol photon m^−2^ s^−1^ to mimic the growth conditions. The quantum yield was based on the formula *Y*(*II*) = (Fm′ − F)/Fm′ [47].

### Fluorescence microscopic analyses

To examine the accumulation of lipid and hydrocarbon contents in *B. braunii* 779 cells, 4 μl of 0.25 mg/ml Nile red in acetone was added to 1 ml of fresh culture according to a previously described method [22]. In brief, after incubation at RT in dark for 15 min, fluorescence signals in the cells were examined using the Zeiss Axiovert 200 M (Carl Zeiss AG, Oberkochen, Germany) with a Zeiss EC Plane Neofluar 40x/0.75 objective lens with a filter cube no. 15 (EX BP 546/12, BS FT 580, EM LP 590). The images were captured by a CoolSNAP HQ monochrome digital camera (Roper Scientific, Ottobrunn, Germany) and processed using MetaMorph software (Molecular Devices, Sunnyvale, CA, USA).

### Analysis of lipid and hydrocarbon contents in *B. braunii* 779 cells

Total lipids and hydrocarbons were extracted using methanol/chloroform (2:1 by volume) and hexane solutions, respectively. In brief, cell samples taken from cultures at various time points were harvested by centrifugation. The resulting cell pellet was resuspended in 0.5 ml PBS buffer (10 mM Na_2_HPO_4_/1.8 mM K_2_PO_4_/137 mM NaCl/2.7 mM KCl, pH7.4) and mixed with 0.5 g of the 0.5 mm acid-washed glass beads (Cat no. G2968, Sigma-Aldrich, St. Louis, MO, USA). Cells were subsequently broken by 6 bursts using a glass-bead beater (MP Biomedicals). Each burst was set for 30 s and a 2-min interval was set between bursts. Broken cells were mixed with 10 volumes of methanol/chloroform (2:1 by volume) or hexane overnight. Organic phase was separated from cell debris and glass beads by centrifugation and transferred into a fresh tube. The tube was subsequently placed in a fume hood with a stream of nitrogen gas to evaporate the solvent. The resulting lipids or hydrocarbons were quantified gravimetrically using the AG204 balance (Mettler-Toledo Inc.).

The lipids were dissolved in chloroform at a concentration of 0.1 mg/μl. Equal amount of lipid was loaded on silica TLC plate (60F254, Merck Corporate, Whitehouse Station, NJ, USA) and developed in hexane/diethyl ether/acetic acid (35:15:0.1 by volume). TAG (tri-oleic acid (C18:1, [cis-9]) glyceride) and FFA (oleic acid (C18:1, [cis-9])) (Sigma-Aldrich, St. Louis, MO, USA) and others were used as standard. Lipid profile on TLC plate was visualized using iodine vapor. The hydrocarbons were dissolved in hexane at a concentration of 0.1 mg/μl and analyzed using Shimadzu GC/MS-QP2010 Plus system (Shimadzu, Kyoto, Japan) equipped with an HP-5 ms Ultra Inert column (30 m × 0.25 mm × 0.25 μm, Agilent Technologies, Santa Clara, CA, USA).

### 18S rDNA sequence-based phylogenetic analysis

Primer sequences (CV1: 5′-TACCTGGTTGATCCTGCCAGTAG-3′; CV2: 5′-CCAATCCCTAGTCGGCATCGT-3′; CV3: 5′-AGATACCGTCGTAGTCTCAACCATAA-3′; CV4: 5′-ACCTTGTTACGACTTCTCCTTCCTC-3′) used in the study of 18S rDNA in several *B. braunii* strains [32] were applied in this study, namely the full length sequence of the 18S rDNA were amplified in two fragments: the left fragment of ~1.0 Kb by primers CV1 and CV2 and right fragment of ~0.8 Kb by primers CV3 and CV4 (see Additional file 11: Figure S2). Sequences of the left and right fragment were assembled into the full length of 18S rDNA of the *B. braunii* 779 with an accession number of BankIt1827606 in NCBI nucleotide database. The *B. braunii* 779 18S rDNA sequence with others (obtained from treeBASE at http://www.treebase.org) was aligned using ClustalX software with Blosum matrix and the phylogenetic tree was obtained using the Bootstrapped Neighbor Joining tree method [48, 49].

### Next-generation sequencing analysis

Total RNA was extracted from *B. braunii* 779 cells prior to and after ND using TRIzol Plus RNA Purification System (Invitrogen-LifeTechnologies Co., Carlsbad, CA, USA) according to manufacturer’s protocol. Approximately 4 μg of the resulting total RNA was used for synthesis of cDNA using the TruSeq RNA Sample Prep Kit (Invitrogen-Life Technologies Co.) according to manufacturer’s instruction including synthesis of first- and second-strand cDNA, end repair, 3′-end adenylation, adapter ligation, fragment enrichment (e.g., ~260 bps in length), and library validation, quantification, and quality assessment with a bioanalyzer (Agilent Technologies; Santa Clara, CA, USA). The libraries are sequenced using the Illumina HiSEQ 2000 Sequencer (BGI, Shenzhen, China).

Over 2 Giga-base clean paired-end reads (90 bps in length/read) from each library were generated using HiSEQ 2000 technology. A total of 55 million reads from both growth conditions were pooled and subjected to de novo assembly using the trinity software [33] (http://trinityrnaseq.github.io) (for detailed description, see Additional file 12). As a result, ~60 thousand non-redundant contigs/scaffolds/ESTs/cDNA (EST was used in this study for simplicity) were obtained based on a cutoff of EST length >300 base pairs and read count >40 per EST. To compare transcription levels in different growth conditions, read counts per EST and its normalized level of FPKM (Fragments Per Kilobase per Million mapped reads) were generated using RSEM software [44]. ESTs with at least one 0 FPKM in 4 measurements were filtered out in this analysis. Deferentially transcribed ESTs upon ND is obtained using EdgeR software [45, 50] based on the raw counts per EST generated by RSEM software [44] using a cutoff of fold change >2 and p value <0.05.

### Annotation of the *B. braunii* 779 transcriptome

All non-redundant ESTs were subjected to sequence homology comparison using the Basic Local Aliment Search Tool BLASTX suit against the pool of “best” proteins in 6 comprehensively annotated microalgae genomes: *Coccomyxa subellipsoidea* C-169 v2 [34], *Chlorella variabilis* NC64A v1 [35], *Chlamydomonas reinhardtii*v4 [36], *Micromonas pusilla* RCC299 v3 [37], *Ostreococcus lucimarinus* v2 [38] and *Thalassiosira pseudonana* CCMP 1335 v3 [39] (genome. jgi-psf.org). A total of 12,292 ESTs showed to share homology to at least a best-hit (i.e., expectation value <1E−07, length of homology to subject/full length >40 %) in the pool of “best” proteins of the 6 genomes. Of 12,292 ESTs, 8,888 (or 72.3 %) and 3,368 (or 27.4 %) were found to be associated with at least one GO function and KEGG ortholog (i.e., expectation value <1E−06), respectively (see Additional file 2: Table S2, Additional file 4: Table S3).

### Pathway-based analysis for potentially co-transcribed subsets of ESTs

We assumed that a subset of co-regulated ESTs that were associated with a KEGG metabolic pathway or GO biological process would exhibit the coherent expression or similar transcription level (i.e. based on rank by level) in log-phase cells or transcription ratio (i.e., based on rank by ratio) in nitrogen-starved cells. To identify the subset of potentially co-regulated ESTs, we applied a sliding window of 1,024 consecutive ESTs in size and 512 ESTs in step for finding subsets of ESTs of a pathway whose occurrence density is twofold higher than that of background with a p value of less than 0.05 after Bonferroni correction [40, 41].

### Statistical analyses

Binomial test was used to determine the statistical significance of enrichment for pathway-associated ESTs (i.e., success) that are present within a window of ranked ESTs (i.e., trials). Bonferroni correction [40, 41] is applied in all multiple tests in this study.

The raw HiSEQ 2000 paired-end sequencing data, the non-redundant transcriptome data, and the annotated transcriptome data generated in this study are available at the NCBI’s GEO database (http://www.ncbi.nlm.nih.gov/geo) with an accession number Series GES71296 (including Platform GPL20730, Samples GSM1831893-96, RSA SPR058734, GES71296 supplemental tables).

## Additional files

Additional file 1:
**Table S1.** List of the 12041 annotated ESTs in *B. braunii* 779. Additional file 2:
**Table S2.** List of GO terms and number of ESTs associated. Additional file 3:
**Figure S1.** Ten GO cellular component (CC) and molecular function (MF) categories associated with the most number of ESTs. Additional file 4:
**Table S3.** List of KEGG pathways and number of ESTs associated. Additional file 5:
**Tables S4–7.** List of homologous ESTs between *B. braunii* 779 and other strains. Additional file 6:
**Tables S8–11.** List of homologous ESTs between *B. braunii* Showa and other trains. Additional file 7:
**Table S12.** List of the differentially transcribed ESTs. Additional file 8:
**Table S13.** List of *B. braunii* 779 enzymes involved in VLCFA biosynthesis based on the transcriptome of *B. braunii* Bot-88. Additional file 9:
**Table S14.** List of *B. braunii* 779 enzymes involved in botryococcene or squalene biosynthesis based on the curated transcriptome of *B. braunii* Showa. Additional file 10:
**Table S15.** List of *B. braunii* 779 enzymes involved in botryococcene or squalene biosynthesis based on the machine-assembled transcriptome of *B. braunii* Showa. Additional file 11:
**Figure S2.** Sequence analysis of the 18S rDNA in *B. braunii* 779. Additional file 12: Supplemental Methods. Detailed steps of transcriptome analysis.

## Abbreviations

CDW: cell dry weight
FPKM: fragments per kilobase per million mapped reads
GC–MS: gas chromatography-coupled mass spectrometry
ND: nitrogen deprivation
TLC: thin layer chromatography
VLCFA: very long chain fatty acid
