# Supplementary material for: Transcriptomic analysis of a moderately growing subisolate Botryococcus braunii 779 (Chlorophyta) in response to nitrogen deprivation
Source: Biotechnol Biofuels. 2015 Aug 28;8:130. doi: 10.1186/s13068-015-0307-y (PMC4552190; doi:10.1186/s13068-015-0307-y)
Supplement: Additional file 3: — Figure S1. Ten GO cellular component (CC) and molecular function (MF) categories associated with the most number of ESTs. [file 13068_2015_307_MOESM3_ESM.pdf]

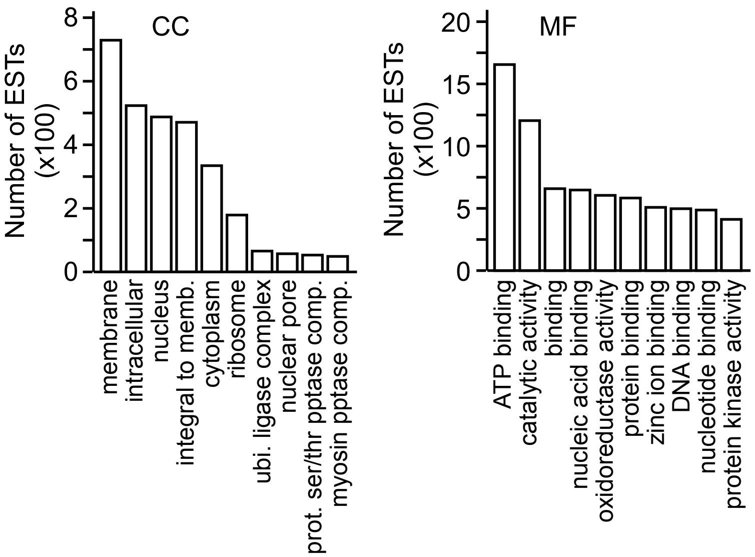

Figure S1. Ten GO cellular component (CC) and molecular function (MF) categories associated with the most number of ESTs. The display is identical to Figure 3D.
