# Supplementary material for: Transcriptomic analysis of a moderately growing subisolate Botryococcus braunii 779 (Chlorophyta) in response to nitrogen deprivation
Source: Biotechnol Biofuels. 2015 Aug 28;8:130. doi: 10.1186/s13068-015-0307-y (PMC4552190; doi:10.1186/s13068-015-0307-y)
Supplement: Additional file 11: — Figure S2. Sequence analysis of the 18S rDNA in B. braunii 779. [file 13068_2015_307_MOESM11_ESM.pdf]

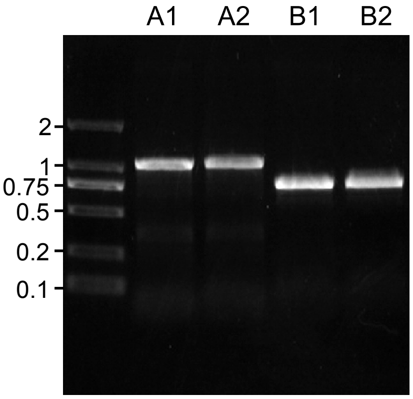

Figure S2. Sequence analysis of the 18S rDNA in *B. braunii* 779. PCR fragments A and B in duplicate were synthesized using primer pairs of CV1/CV2 and CV3/CV4 on genomic DNA of *B. braunii* 779. The fragments were subjected to nucleotide sequence determination.
