# Supplementary material for: Transcriptomic analysis of a moderately growing subisolate Botryococcus braunii 779 (Chlorophyta) in response to nitrogen deprivation
Source: Biotechnol Biofuels. 2015 Aug 28;8:130. doi: 10.1186/s13068-015-0307-y (PMC4552190; doi:10.1186/s13068-015-0307-y)
Supplement: Additional file 12: — Supplemental Methods. Detailed steps of transcriptome analysis. [file 13068_2015_307_MOESM12_ESM.docx]

**Additional file 12**

**Supplemental Methods for transcriptome analysis**

**Short-read sequencing files used in transcriptomic analysis**

Short-read sequencing data in biological duplicate were generated by using the Illumina Hiseq 2000 paired-end (PE90) method in BGI. Each sample generates 2G good-quality sequences. Name of the data files are listed as follows:

Samples prior to ND:

1. 779_12-19_I1_1.fq and 779_12-19_I1_2.fq

2. BOTgolTABRAAPEI-46_L7_1.fq and BOTgolTABRAAPEI-46_L7_2.fq

(or 779-1_12-27_L7_1.fq and 779-1_12-27_L7_2.fq)

Samples after ND:

3. 779N12-10_I1_1.fq and 779N12-10_I1_2.fq

4. 779N12-22_I1_1.fq and 779N12-22_I1_2.fq.

Sample sequences 1 and 3 were pooled for de novo transcriptome assembly and all 1-4 sample sequences were used for estimation of read count per ESTs and normalized level of FPKM.

**Assembly of the non-redundant transcriptome of B. braunii 779**

Sample sequences were first subjected to quality control analysis using FastQC (ver 0.11.2). Based on the analysis, we removed 12 nucleotides at the 5’-end in all reads and 5 nucleotides at the 3’-end for some read when necessary. The minimum length of the output reads was set to 36 nucleotides using Trimomatic (ver 0.32). The resulting reads were subsequently used for de novo transcriptome assembly using Trinity (ver 20140717) with the minimal contig length of 300 nucleotides, which generate 138,295 sequence contigs. All contigs were subject to analysis for removing redundant sequences using CD-Hit. After filtering the redundant contigs (i.e., identity > 90%) and low read-counts contigs (i.e., counts per contig < 40), a total number of 61,220 contigs or ESTs (i.e., ESTs were used hereafter) were obtained and designated as non-redundant transcriptome (i.e., available in GEO database with an accession number of series GSE71296). The statistic of the non-redundant transcriptome is as follows:

Contig N10: 2835

Contig N20: 2016

Contig N30: 1557

Contig N40: 1229

Contig N50: 985

Median contig length: 609

Average contig length: 836.65

Total assembled bases: 51219717

Percent GC: 53.36

**Estimation of EST transcription levels**

Individual reads were aligned to the non-redundant transcriptome using Bowtie2. The aligned reads were subjected to estimation of read count and normalized level (FPKM) using RSEM. The read counts were subjected to analysis of differentially transcribed ESTs identification using EdgeR with a cutoff of fold-change > 2, p-value < 0.05). Information related to the EST normalized levels and differential expression (i.e., fold change and p-value) (see series GSE71296 in GEO database).

Normalization factors used in EdgeR analysis is listed as follows:

group desc. lib.size norm.factors

0320_779_12-19 +N_rep1 1088168 1.045

0320_779-1_12-27 +N_rep2 1204890 1.050

0320_779N12-10 -N_rep1 1263690 1.166

0320_779N12-22 -N_rep2 1270455 0.781

**Annotation of the non-redundant transcriptome**

Non-redundant transcriptome was subjected to sequence homology search against the “best” protein database consistent of 6 comprehensively annotated genomes: *Coccomyxa subellipsoidea* C-169 v2 {Blanc, 2012 #302}, *Chlorella variabilis* NC64A v1{Blanc, 2010 #15}, *Chlamydomonas reinhardtii*v4{Merchant, 2007 #14}, *Micromonas pusilla* RCC299 v3 {Worden, 2009 #306}, *Ostreococcus lucimarinus* v2 {Palenik, 2007 #307} and *Thalassiosira pseudonana* CCMP 1335 v3 {Armbrust, 2004 #308} (genome. jgi-psf.org) using BLASTX method. EST best-hits were obtained based on filters of e-value 1E-07 and length of homologous sequences versus full length was greater than 40%. The best-hit-associated GO and KEGG annotations were applied to the respective ESTs. Non-expression ESTs were also filtered out based on the condition of having one of the 4 samples whose FPKM level was 0. To this end, a set of 12,292 transcribed and annotated ESTs were obtained and subsequently subjected to various transcriptional profiling analyses in this study (see Additional file 1).

**Pathway-based analysis in log-phase growth cells and in response to ND**

Pathway-based analysis is to determine the enrichment of the pathway-associated ESTs in a moving window of 1,024 ESTs in size and 512 ESTs in steps alone the ranked ESTs by level (i.e., average FPKM level in cells prior to ND) and ratio (i.e., ratio of average FPKM levels between cells after and prior to ND). Randomly selected 4 EST without GO or KEGG association were removed from the pathway-based analysis to allow covering all 12,288 ESTs (i.e., = 12,292-4) in the moving window analysis. GO biological processes and KEGG metabolic pathways whose associated EST in transcriptome is 30 or greater are considered for this analysis. To this end, 44 GO biological processes and 59 KEGG metabolic pathways were used in this analysis and all p-values were adjusted by multiplying a factor of 105 (i.e., correction factor also included the addition of ribosome and differentially transcribed ESTs in this multiple test) or 105*p-value < 0.05. The enrichment is based on density $D_{wi}=N_{wi}/1024$over average density $D_{ave}=N_{all}/12,288$, where $D_{wi}$ is the density in window *i*.

**Some software settings used in software for analysis of the transcriptome in this study**

*Trinity for assembly of transcriptome*

Usage: --JM 110G --SS-lib_type FR –CPU_threads 60 --min_contig_length 300

where JM 100G stands for 110GB memory allocated, SS_lib_type FR for paired-end of sequencing library, CPU_threads 60 for the CPU capacity of the machine processor, min_contig_length 300 for minimal contig length of 300 nucleotides.

*RSEM (Trinity perl script: align_and_estimate_abundance.pl)*

Usage: --est_method RSEM --aln_method bowtie2 --SS_lib_type FR --thread_count 60

where reads are aligned to the non-redundant EST sequences using bowtie2 and read count per EST is estimated by RSEM.

*EdgeR (Trinity perl script: abundance_estimates_to_matrix.pl and run_DE_analysis.pl)*

Based on the read count per EST, EdgeR output the fold change (in log2 scale), p-value, and false discovery rate (FDR). Differentially expressed ESTs are defined as those whose fold change > 2 and p-value < 0.05 (see Additional file 7).

*BLASTX (v2.2.30) for comparison of homologous sequences*

Usage: -max_target_seqs 1 -num_threads 60 -outfmt 6 -evalue 1e-7

where max_target_seqs 1 stands for a best-hit, num_threads 60 for machine’s CPU cappacity, outfmt 6 for type of output format, evalue 1e-7 for cutoff setting.
